# High detection rate of osteoporosis with screening of a general hospitalized population: a 6-year study in 6406 patients in a university hospital setting

**DOI:** 10.1186/s12891-020-3116-9

**Published:** 2020-02-10

**Authors:** Olivier Malaise, Marie Detroz, Mathieu Leroy, Lorenzo Leonori, Laurence Seidel, Michel G. Malaise

**Affiliations:** 1 Departments of Rheumatology, University Hospital of Liège, Liège University, Liège, Belgium; 2 Departments of Biostatistics, University Hospital of Liège, Liège University, Liège, Belgium

**Keywords:** Osteoporosis, Dual energy X-ray absorptiometry, Hospitalization, Male

## Abstract

**Background:**

Osteoporosis is a highly prevalent disease identified by Dual Energy X-ray Absorptiometry (DEXA) that can be performed in an ambulatory (out-patient) or hospitalized population. We evaluated the use of baseline in-hospital DEXA screening to identify osteoporosis in ambulatory care and hospitalized patients; we also assessed specific risk factors for osteoporosis among these populations.

**Methods:**

We included a baseline initial DEXA from 6406 consecutive patients at our tertiary referral University Hospital.

**Results:**

Osteoporosis was diagnosed in 22.3% of the study population. In univariate analysis, osteoporosis risk factors were age, fracture history and low BMI (for all 3 sites), but also corticotherapy (lumbar spine and femoral neck) and male (lumbar spine). In multivariate analysis, age, fracture history, low BMI, and male increased osteoporosis risk. In-hospital screening yielded a higher percentage of osteoporosis positive scans than ambulatory care screening (31.8% vs 18.5%, *p* < 0.001). In-hospital screening targeted an older and more predominantly male population with a higher fracture history. Z-scores revealed that this difference was not only due to an older age of the population and mainly concerned cortical bone.

**Conclusions:**

In-hospital osteoporosis screening revealed more osteoporosis than screening in ambulatory practice and could be an additional tool to improve the identification and management of osteoporosis. In addition to typical risk factors, we identified male gender as associated with osteoporosis detection in our cohort.

## Background

Osteoporosis is a disease characterized by low bone mass and deterioration of bone micro-architecture, leading to fragility and an increased fracture risk. Osteoporosis has a high prevalence in women in industrialized countries, ranging from 9 to 15% based on total hip bone mineral density (BMD) and 16–38% when vertebral BMD was included [1]. Osteoporosis also affects males, albeit at a decreased rate, with a global prevalence of 3% (hip) and 8% (vertebra) [1]. Between 2005 and 6 and 2013–14, there was a decline in bone mass density in older US adults, indicating that osteoporosis prevalence is not decreasing and is far from being resolved [2]. BMD is strongly correlated to fracture risk [3]. The risk of vertebral or femoral neck fracture was 18.0 and 28.0 times higher, respectively, when women after 50 years old were osteoporotic [4]. Ten to 20 % of patients with hip fracture died in the first year following the fracture with a risk of premature mortality that remains elevated for at least 10 years [5, 6].

Osteoporosis is preventable and treatable using the various pharmacological therapies available and this management reduces fracture rates [7, 8]. Dual-Energy X-ray Absorptiometry (DEXA) scan is the cornerstone of osteoporosis screening, while the Fracture Risk Assessment Tool (FRAX) score adds valuable further information when evaluating fracture risk [9, 10]. Despite the clear links between osteoporosis and fracture, screening rates are low in primary care with only 21.1, 26.5 and 12.8% of women aged 50–64, 65–79 and > 80 years, respectively, being screened in a large US study [5], while only 10.8% of women > 65 years underwent osteoporosis screening in a US primary care setting [11]. Even after hip fracture, the screening rate remains low with only 17.1 and 23.1% of women undergoing osteoporosis assessment/treatment within 6 and 12 months of their fracture, respectively [12]. Data from other countries also confirm the lack of osteoporosis screening [13]. As a consequence of this lack of primary and secondary prevention, emphasis has recently been placed on improved screening efforts after an initial fracture event, such as the development of “fracture liaison services”, that are effective in reducing second fractures [14]*.* To improve osteoporosis screening, opportunistic use of CT scan has also been proposed to analyze BMD [15] and detect osteoporotic fractures [16], as well as FRAX algorithm utilization [17].

As a population, hospitalized patients have variable comorbid conditions and receive pharmacotherapies that can have deleterious effects on bone thereby leading to osteoporosis. Targeting such hospitalized patients could represent an opportunity to improve the detection of osteoporosis. Hence, we studied the effectiveness of in-hospital osteoporosis screening using DEXA (T and Z-scores: lumbar spine; femoral neck, total hip) and risk factor scores, and compared the osteoporosis rate in a hospitalized sub-population with that of patients screened in the ambulatory care setting.

## Methods

### Patients

The Department of Rheumatology of the University Hospital of Liège (Belgium) offers DEXA studies to ambulatory out-patients prescribed by general practitioners or by medical specialists during clinic-based consultation or to hospitalized in-patients in various departments (e.g. rheumatology, neurology, internal medicine, endocrinology …).

### DEXA procedure

All the examinations were performed by the same Discovery A DEXA system (Hologic®, Bedford, MA, USA), with lumbar spine (L1-L4), total hip and femoral neck analysis. For total hip and femoral neck, the left side was analyzed except when prosthetic material was present. T-scores and Z-scores were reported for these three sites. T-score values were considered as normal if > − 1, osteopenic if ≤ − 1 and > − 2.5 and osteoporotic if ≤ − 2.5. Z-score were also categorized in different ranks: < − 1, <− 2 and ≤ − 2.5. The proportion of patients with normal values, osteopenia or osteoporosis was analyzed for each year and for the global period (2007–2012). The same specialized nurse (ML) or physiotherapist (LL) is in charge of the DEXA determination, whatever the origin of the patients. Standardization procedures were performed according to the International Society for Clinical Densitometry. In particular, during the years analyzed, daily quality control with phantom were performed to ensure that these values were located at maximum +/− 1.5% of the mean value of calibration. About the parameters of the DEXA (Hologic®, Bedford, MA, USA), the total bone mineral density (BMD) coefficient of variation (CV) = 1.0%.

### Study protocol

We conducted a retrospective study to analyse our DEXA results in real-life conditions. Approval was obtained from the local ethical committee of the University Hospital of Liège (reference 2019/152). We only considered DEXA scan that were realized our Discovery A DEXA system in our instution, and not DEXA scans that could be realized in another center. Data were available for the 2007–2012 period and 9354 DEXA scan were performed in our hospital during that period. The only specific exclusion criteria was age < 18 years. During the period analyzed, only the patient’s initial DEXA study was assessed and subsequent DEXA scans were not included. After application of these criteria, 6406 first DEXA examinations were included in the study database. Data concerning sex, age, body mass index (BMI) and history of previous fracture (whatever the site) were collected. Medication data included history of glucocorticoid (GC) use as defined by FRAX (current or previous treatment for > 3 months at a prednisone dose ≥5 mg/day) and proton-pump inhibitors (PPI) use. All data was de-identified in the study database: data will be made anonymous as soon as it is encoded and stored in a locked cabinet. Only the investigators of the study will have access to the data collected.

To assess whether any differences existed in terms of the diagnosis rates for osteoporosis, we divided our study population (*n* = 6406 patients) in two groups: ambulatory care and hospitalized in-patients.

### Statistical analysis

Results were presented as mean ± standard deviation (SD) or as median and range (minimum-maximum) for continuous variables and as frequency tables for qualitative variables. Comparisons between ambulatory and hospitalized patients were performed using Student’s t-test for continuous variables and the chi-square test for the categorical variables. Univariate logistic regression models investigated the relationship between osteoporosis risk and demographic variables (age, BMI, sex GC use, PPI intake and previous fracture). Multivariate logistic regression model was applied on these factors and the year of the DEXA scan. The results were considered significant at the uncertainty level of 5% (*p* < 0.05). Calculations were performed using SAS software version 9.4 (SAS Institute, Cary, NC, USA).

## Results

### Demographics

Six thousand four hundred six first DEXA examinations were included (2007: *n* = 1494; 2008: *n* = 1158; 2009: *n* = 1079; 2010: *n* = 945; 2011: *n* = 868; 2012: *n* = 862). In the 6406 patients that had a first DEXA scan, the mean ± SD (min, max) age was 60.5 ± 14.3 (18, 98) years and the mean ± SD (min, max) BMI was 25.2 ± 5.1 (11.7, 60.4) kg/m^2^. In total, 74.4% of patients were female. Overall, 30.5% of the patients had a history of GC use and 28.7% were taking a PPI. A previous fracture was recorded in 29.0% of patients. At the time of DEXA examination, 4561 patients (71.2%) were ambulatory and 1845 (28.8%) patients were hospital in-patients.

### DEXA results

Normal, osteopenia and osteoporosis rates in the overall study population are shown in Table 1. The median (min, max) T-scores were − 1.0 (− 6.0, 8.0) for lumbar spine, − 1.3 (− 5.2, 3.8) for femoral neck and − 1.0 (− 5.2, 6.0) for total hip, respectively. Osteoporosis at least one of the three sites was identified in 1429/6406 patients (22.3%). At each anatomical location osteoporosis rates were as follows: 13.7% at the lumbar spine; 13.6% at the femoral neck and 9.2% at the total hip (Table 1). Lastly, 252 of 6406 patients (3.9%) had osteoporosis at all the three sites (Table 1).

**Table 1:** Osteoporosis diagnosis in the whole population (2007–2012)

	Categories	*N*	Number (Percent)
Global diagnostic		6406	
	Normal		1724 (26.9)
	Osteopenia		3253 (50.8)
	Osteoporosis		1429 (**22.3**)
Lumbar spine diagnostic		6377	
	Normal		3121 (48.9)
	Osteopenia		2380 (37.3)
	Osteoporosis		876 (**13.7**)
Femoral neck diagnostic		6262	
	Normal		2277 (36.4)
	Osteopenia		3134 (50.0)
	Osteoporosis		851 (**13.6**)
Total hip diagnostic		6278	
	Normal		3033 (48.3)
	Osteopenia		2668 (42.5)
	Osteoporosis		577 (**9.2**)
Osteoporosis at the three sites		6406	
	No		6154 (96.1)
	Yes		252 (**3.9**)

**Fig. 1:**
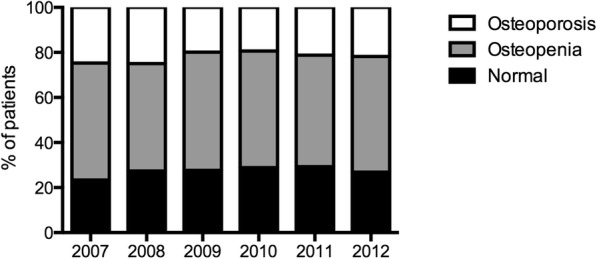
Percentage of normal, osteopenic and osteoporotic patients according to the year of DEXA examination for the whole population

Overall, patients with normal T-scores, osteopenia or osteoporosis on DEXA had a statistically different mean age (SD) of 56.4 (13.9), 60.6 (13.8) and 65.3 (14.4) years old, respectively (*p* < 0.001 for each comparison). Figure 1 shows the percentage of normal, osteopenic and osteoporotic patients according to the year of DEXA examination for the whole population. The percentage of osteoporotic patients changed according to a quadratic model with time (linear term *p* = 0.0025, quadratic term: *p* = 0.022) with variations from year to year; the percentage of osteopenic patients remained stable (*p* = 0.95) throughout the study period (Fig. 1).

### Osteoporosis risk factors

In univariate analysis for the whole population (*n* = 6406), risks factor for osteoporosis (independently of the anatomical site, i.e. T-score ≤ − 2.5 at the lumbar spine, total hip or femoral neck) were older age, a lower BMI, GC intake and a previous fracture (Table 2). The risk factors were also described separately for each anatomical site: older age, lower BMI and a previous fracture were associated with osteoporosis at all three anatomical sites, while GC intake was only correlated with lumbar spine and femoral neck. Female was associated with osteoporosis detection at the total hip, and male for the lumbar spine (Table 2).

**Table 2:** Risk factors for osteoporosis in univariate (left) and multivariate (right) analysis in the whole population (2007–2012)

	OR	IC95%	*p*-value	OR	IC95%	*p*-value
Global analysis						
Age (years)	1.03	1.03–1.04	**< 0.0001**	1.03	1.02–1.03	**< 0.0001**
BMI (kg/m^2^)	0.88	0.87–0.90	**< 0.0001**	0.86	0.84–0.87	**< 0.0001**
Sex (male)	1.12	0.98–1.28	0.094	1.23	1.02–1.47	**0.029**
GC use	1.20	1.06–1.36	**0.0051**	1.18	0.98–1.41	0.084
PPI intake	0.99	0.85–1.17	0.95	0.97	0.81–1.17	0.77
Previous fracture	3.20	2.83–3.62	**< 0.0001**	2.80	2.36–3.32	**< 0.0001**
Lumbar spine						
Age (years)	1.02	1.01–1.02	**< 0.0001**	1.01	1.00–1.02	**0.010**
BMI (kg/m^2^)	0.91	0.90–0.93	**< 0.0001**	0.90	0.88–0.92	**< 0.0001**
Sex (male)	1.52	1.30–1.77	**< 0.0001**	1.78	1.45–2.18	**< 0.0001**
GC use	1.18	1.01–1.37	**0.032**	1.06	0.86–1.31	0.60
PPI intake	0.91	0.74–1.11	0.35	0.90	0.72–1.11	0.33
Previous fracture	2.56	2.22–2.97	**< 0.0001**	2.47	2.03–3.02	**< 0.0001**
Femoral neck						
Age (years)	1.05	1.04–1.06	**< 0.0001**	1.05	1.04–1.06	**< 0.0001**
BMI (kg/m^2^)	0.84	0.83–0.86	**< 0.0001**	0.79	0.77–0.82	**< 0.0001**
Sex (male)	0.87	0.74–1.04	0.12	0.90	0.71–1.15	0.39
GC use	1.20	1.03–1.40	**0.019**	1.31	1.03–1.65	**0.025**
PPI intake	1.08	0.89–1.32	0.43	1.09	0.87–1.37	0.47
Previous fracture	3.63	3.13–4.21	**< 0.0001**	2.95	2.38–3.66	**< 0.0001**
Total hip						
Age (years)	1.05	1.04–1.06	**< 0.0001**	1.04	1.03–1.05	**< 0.0001**
BMI (kg/m^2^)	0.82	0.80–0.84	**< 0.0001**	0.78	0.75–0.80	**< 0.0001**
Sexe (male)	0.68	0.55–0.85	**0.0004**	0.87	0.65–1.16	0.33
GC use	1.13	0.94–1.36	0.20	1.19	0.89–1.57	0.24
PPI intake	0.90	0.70–1.15	0.38	0.87	0.65–1.15	0.32
Previous fracture	4.58	3.83–5.48	**< 0.0001**	3.79	2.92–4.92	**< 0.0001**

A multivariate analysis for the global osteoporosis risk included seven variables: year of the DEXA scan, age, BMI, sex, GC use, PPI intake and previous fracture. This analysis included 4090 of the initial population with complete data for all variables. In this multivariate analysis we observed significant effects of age [odds ratio (OR), (95% confidence interval): 1.03 (1.02–1.03), *p* < 0.0001], BMI [OR: 0.86 (0.84–0.87), *p* < 0.0001], male [1.23 (1.02–1.47), *p* = 0.029] and an history of a previous fracture [2.80 (2.36–3.32), *p* < 0.0001]. Age, BMI and fracture were significant risk factors for each anatomical site separately [OR (IC95%) at lumbar spine: 1.01 (1.00–1.02), 0.90 (0.88–0.92), 2.47 (2.03–3.02) respectively; OR at femoral neck: 1.05 (1.04–1.06), 0.79 (0.77–0.82), 2.95 (2.38–3.66) respectively; OR total hip1.04 (1.03–1.05), 0.78 (0.75–0.80), 3.79 (2.92–4.92) respectively (*p* < 0.0001 for all expect for age and lumbar spine with *p* = 0.01)]. GC use was significant associated with OP at the femoral neck [OR 1.31 (1.03–1.65), *p* = 0.025] and male with OP at lumbar spine [OR 1.78 (1.45–2.18), *p* < 0.0001]. These data were described in Table 2.

### Comparisons between ambulatory and hospitalized patients

On the 6406 patients, 4561 (71.2%) were ambulatory and 1845 (28.8%) were hospitalized. In-patients originated from rheumatology (21.8%), neurology (15.5%), internal medicine (14.7%), endocrinology (11.8%), orthopedic surgery (5.7%), nephrology (5.5%), pulmonary (4.4%), cardiology (4.0%), neurosurgery (3.8%) and abdominal surgery (3.4%) representing 90.6% of the cohort (77.7% from medical departments and 12.9% from surgical departments). Percentage of osteoporotic patients by department in the hospitalized sub-population was represented in Additional file 1: Figure S1, with the highest percentage for the pneumology department. T-score [median (inter-quartile range)] at the lumbar spine, the femoral neck and the total hip were significantly lower among the hospitalized patients. At each anatomical site the T-scores were as follows: lumbar spine: − 1.0 (− 1.9; 0.1) vs. -1.1 (− 2.1; 0.0) (*p* = 0.0001); femoral neck − 1.2 (− 1.9; − 0.5) vs. -1.5 (− 2.3; − 0.7) (*p* < 0.0001) and total hip − 0.9 (− 1.6; − 0.2) vs. -1.3 (− 2.1; − 0.5) (*p* < 0.0001) in the ambulatory and in-patient groups, respectively.

**Fig. 2:**
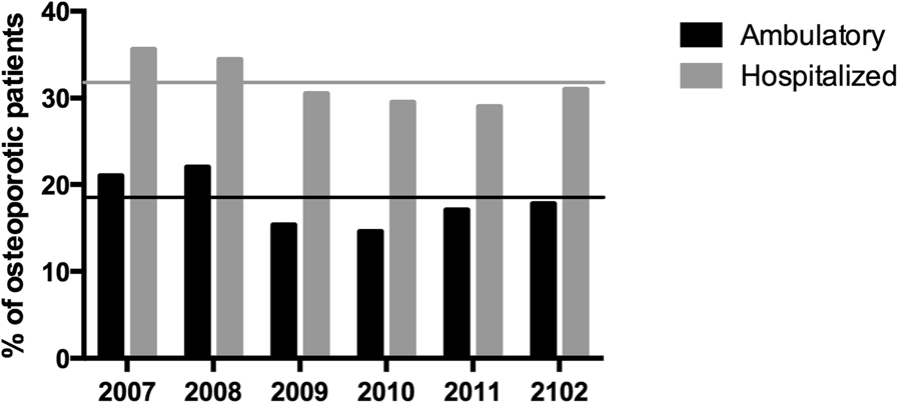
Comparison between the percentage of osteoporotic patients by year in ambulatory and hospitalized patients. The grey horizontal line represented the 2007–2012 mean percentage in the hospitalized sub-population. The black horizontal line represented the percentage in the ambulatory sub-population

For the diagnosis of osteoporosis, this was present at at least one site in 18.5% of the ambulatory patients, but this rose significantly to 31.8% in the hospitalized population (< 0.0001) (Table 3). Lumbar spine, femoral neck and total hip were also separately analyzed, with significantly more osteoporosis diagnosed for each site in the in-patient group (lumbar spine (%): 11.4 vs 19.1; femoral neck 10.4 vs 21.5; total hip 6.2 vs 16.6 for ambulatory and hospitalized patients respectively, all *p* < 0.0001). A total of 2.3% of the ambulatory patients had osteoporosis at all the three sites, while this percentage was nearly 3.5 times higher (7.9%) (*p* < 0.0001) in hospitalized patients. The differences in osteoporosis diagnosis between ambulatory and hospitalized patients over each year remained stable throughout the analysis period (Fig. 2).

**Table 3:** Osteoporosis diagnosis in ambulatory and hospitalized patients

		Ambulatory patients		Hospitalized patients		*p*-value
		*N*	Number (%)	*N*	Number (%)	
Global osteoporosis		4561		1845		**< 0.0001**
	No		3719 (81.5)		1258 (68.2)	
	Yes		842 (**18.5**)		587 (**31.8**)	
Lumbar spine osteoporosis		4541		1836		**< 0.0001**
	No		4022 (88.6)		1479 (80.6)	
	Yes		519 (**11.4**)		357 (**19.4**)	
Femoral neck osteoporosis		4472		1790		**< 0.0001**
	No		4005 (89.6)		1406 (78.5)	
	Yes		467 (**10.4**)		384 (**21.5**)	
Total hip osteoporosis		4484		1794		**< 0.0001**
	No		4205 (93.8)		1496 (83.4)	
	Yes		279 (**6.2**)		298 (**16.6**)	
Osteoporosis at the three sites		4561		1845		**< 0.0001**
	No		4454 (97.7)		1700 (92.1)	
	Yes		107 (**2.3**)		145 (**7.9**)

Demographic data and risk factors for osteoporosis were also different between ambulatory and hospitalized patients (Table 4): hospitalized patients were significantly older (*p* < 0.0001) and more had a previous fracture (*p* < 0.0001). Males were significantly higher in proportion in hospitalized patients (*p* < 0.0001). BMI was also higher in hospitalized patients (*p* = 0.028). There was no difference in terms of history of GC use.

**Table 4:** Demographic and clinical comparison between ambulatory and hospitalized patients

		Ambulatory patients		Hospitalized patients		*p*-value
		*N*	Mean ± SD /Number (%)	*N*	Mean ± SD /Number (%)	
			4561 (71.2)		1845 (28.8)	
Age (years)		4561	**59.0** ± 13.2	1845	**64.2** ± 16.2	**< 0.0001**
BMI (kg/m^2^)		4478	**25.1** ± 4.82	1690	**25.4** ± 5.79	**0.028**
Sex		4561		1845		**< 0.0001**
	F		3571 (78.3)		1196 (64.8)	
	M		990 (**21.7**)		649 (**35.2**)	
GC use		4556		1836		0.078
	No		3136 (68.8)		1305 (71.1)	
	Yes		1420 (31.2)		531 (28.9)	
PPI intake		2910		1341		0.15
	No		2096 (72.0)		937 (69.9)	
	Yes		814 (28.0)		404 (30.1)	
Previous fracture		4555		1837		**< 0.0001**
	No		3419 (75.1)		1120 (61.0)	
	Yes		1136 (**24.9**)		717 (**39.0**)	

To determine whether the difference between T-score was related only to greater age in the hospitalized versus the ambulatory patient group (mean ages 64.2 vs 59.0 years, respectively), Z-scores were also compared. Z-scores at the femoral neck and the total hip were significantly lower (*p* < 0.0001) in hospitalized vs ambulatory patients; Z scores at the lumbar spine were lower in the hospitalized group, but the difference was not significant (*p* = 0.076). When Z-scores were ranked as <− 1, <− 2, <− 2.5, the hospitalized group had significantly lower ranked scores than the ambulatory group (*p* < 0.0001) irrespective of the anatomical site studied (Additional file 2: Table S1). Z-score ranks of <− 1, <− 2 and < − 2.5 were 1.4 to 1.7, 2.1 to 2.6 and 2.3 to 3.9 times more prevalent among hospitalized versus ambulatory patients at the lumbar spine, femoral neck and total hip, respectively (Additional file 2: Table S1).

## Discussion

According to our knowledge, this is the first large study evaluating DEXA screening results in a hospitalized sub-population in comparison to an ambulatory group and demonstrating a higher detection rate of osteoporosis among the in-patient population. Among 6406 patients having a DEXA examination, globally, osteoporosis was detected in 22.3% of cases. This prevalence is in agreement with a recently published prevalence of 16–38% based on lumbar spine and of 9–15% based on total hip BMD [1]. When the cohort was divided into ambulatory (*n* = 4561) and hospitalized (*n* = 1845 patients), we found striking differences, with hospitalized patients having significantly lower T-scores and having with 2–3 times higher rates of osteoporosis at the lumbar spine, femoral neck and total hip evaluations individually, as well as all three sites. This better detection rate in osteoporosis indicated that an in-hospital screening strategy could be an effective additional tool to improve osteoporosis screening and identification.

As we mentioned, DEXA evaluation was not systematic in any of the group but depended on the physician’s judgment. Our study was retrospective and the better detection rate of in-hospital screening could not be due to a selective positive bias. This also revealed the lack of efficient screening in primary care, without any improvement between 2007 and 2012. In another study about osteoporosis screening in inflammatory bowel population, a population at high risk of multifactorial osteoporosis, the screening rate was higher in tertiary referral center compared to secondary center [18]. It’s worlwide recognized that osteoporosis secreening is defective. In an ideal world, an optimal screening would be realized in primary care, with a systematic DEXA-scan for woman after menopausis and earlier if there are risk factor for osteoporosis, as proposed by several international guidelines. For man, the consensus is to do not perform DEXA-scan systematicaly, but only if there is a risk factor. For the in-hospital procedure, the same attitude should be proposed if the ambulatory care did not altready perform it. Hospitalization should be the place to think about osteoporosis screening and to perform it if there were no previous screening in ambulatory care.

Hospitalized patients were significantly older with a higher BMI and with more previous fractures than ambulatory patients. GC use and PPI intake were not different in the two sub-populations. Theoretically, older age and more history of major fractures of hospitalized patients could explain the better efficiency. Of interest, only a minor percentage (5.7%) of hospitalized patients came from the orthopedic surgery department: the difference between hospitalized and ambulatory patients was consequently not due to hospitalization for an acute fracture. As 78% of the cohort came from medicine departments, comorbidities or treatment that we could suppose more numerous in this population could also negatively influenced bone mineral density (inflammatory systemic disease with e.g. rheumatoid arthritis [19], polymyalgia rheumatica [20], systemic erythematous lupus [21]; inflammatory bowel disease [22]; mellitus diabetes [23]; renal failure [24]; lung disease [25]…). Due to this better positive detection rate, emphasis should be put on the in-hospital osteoporosis screening, because it’s the place where much co-morbiditiy accumulate. More systematic screening of the in-hospital population could help to improve osteoporosis detection. In-hospital physicians could also be more concerned or have more time to take care with patients’ comorbidities.

In order to neutralize the age parameter, we also decided to compare Z-scores (without age influence): Z-scores ranking analysis (<− 1, <− 2, <− 2.5) showed significant difference percentage between ambulatory and hospitalized patients in each of the 3 categories and in each of the three sites studied. Z-scores of the femoral neck and of total hip of hospitalized patients were also significantly lower than those of the ambulatory ones, while differences did not reach statistical signification (*p* = 0.076) at the lumbar spine, although lower. Excess of prevalence in hospitalized patients compared to ambulatory ones increased according to the severity of the category (<− 1, <− 2, <− 2.5) and was maximal in the category Z-score < 2.5 with nearly four times more in femoral neck and total hip evaluations compared to twice more in lumbar spine evaluation, confirming that differences affected more cortical than trabecular bone. Even if our data were not enough complete in terms of co-morbidities description for each patients, we can hypothesize that trabecular difference would be more influenced by the age difference between the patients and that the cortical difference could be more a reflect of comorbidities linked to inflammatory pre-existing conditions. A limitation of our study was the incomplete characterization in the comorbidities of our patients, that were restricted to the data that we collected that were presented in the results section. Systematic collection of the comorbidities, the drugs and the medical history should be planned to identify which co-morbidities could be associated to the higher rate of osteoporosis in the hospitalized population. In attempt to answer to that question in the future, specific informatic programs that automatically link DEXA data with identification of comorbidities on both sub-populations are currently under development.

Older age, lower BMI and history of fracture (all three in both univariate and multivariate analysis) were classical risk factors present in our study for osteoporosis diagnostic. GC use was another classical osteoporosis risk factor in the univariate analysis at least in one site as well as at the lumbar spine and femoral neck evaluation. In the multivariate analysis however, signification was restricted to the femoral neck evaluation, which is less usual. Explanation could be that GC use is not causal in this situation, but indirectly reflected co-existence of co-morbidities, justification of their use (chronic GC were prescribed in case of inflammatory disease, such as rheumatoid arthritis, polymyalgia rheumatic, vasculitis … and outside the field of rheumatology with e.g. inflammatory bowel disease, but also after solid or hematopoietic graft or for respiratory disease with asthma or chronic obstructive pulmonary disease). However, we did not have information about dose and duration of GC treatment that could influence the correlation. Male was also associated with OP detection in the univariate analysis at the lumbar spine evaluation. The multivariate analysis confirmed association between male at the lumbar spine but also for osteoporosis diagnostic at least in one of the three sites. Osteoporosis is known to be more common in women and recently it was estimated that women aged 50 years or older have a fourfold higher rate of osteoporosis and a twofold higher rate of osteopenia as compared with men [26]. As a direct consequence, screening was more frequently performed in the female population: e.g. in a primary care center, the screening rate of patients was 60% for female, but only 18.4% for men [27] or only 11% of the eligible men aged 70 years of older in an other study [28]. It must be underlined that there is no real consensus for the screening of men population in the international guidelines. However, male osteoporosis prevalence could be underestimated: a study found a similar prevalence of osteoporosis for men aged 70 years or older and women aged 65 years [27]. After a hip fracture (secondary prevention), osteoporosis screening was also lower in men [29], even if men are known to have a higher rate of complications after an osteoporotic fracture, with a 70% greater risk of mortality compared with women in a Danish registry [30]. In our cohort, the trend was similar: men only represented 25.6% of the total population, rising to 35.2% in the hospitalized population. In univariate analysis, male is associated with osteoporosis at the lumbar spine and this was confirmed in multivariate analysis for the global diagnosis of osteoporosis: men in our population are at greater risk to have osteoporosis than women. An explanation could be that women are more often screened (due to better awareness about osteoporosis risk for women), with less effective screening, whereby only men with established risk factor of osteoporosis were screened. In a study conducted in an inflammatory bowel disease tertiary center, the authors also found that female are more likely to be screened, but male patients were diagnosed more often with osteopenia or osteoporosis than females [31]. Another explanation is that screening is deficient in men leading to underdiagnosis of impaired bone health in men. Recently, an additional paper about patients with inflammatory bowel disease also found that male was a risk factor for osteoporosis [32]. However, in these two papers, osteoporosis was defined as T-score − 2.5 at any of the site studied and there were no analysis for each site separately, as we have performed.

There exist other limitations in our work: the study was retrospective (and we could only rely on information that were available in the patient’s medical record), only compared T-scores between the two sub-populations and did not performed a direct comparison between the bone mass (g/cm^2^). Nevertheless, this is real life and results were unexpected when we started the study.

## Conclusions

In-hospital osteoporosis screening identified more osteoporosis than in ambulatory practice. Promoting this in-hospital detection could be an additional tool to improve osteoporosis managing. This difference is not only due to an older age of the population and suggests the existence of more numerous comorbidities deleterious for bone health in hospitalized patients. In addition to usual risk factors (age, lower BMI, previous fracture), we identify male as associated to osteoporosis detection in our cohort. This underlines the necessity not only to screen women, but also to think about men.

## Supplementary information

**Additional file 1: Figure S1.** Percentage of osteoporotic patients by department in the hospitalized sub-population. The horizontal line represented the mean percentage in the hospitalized sub-population. The percentage in the X axe legend after the name of the department represent the % of DEXA performed by the department regards to the total number of DEXA performed in the hospitalized sub-population.

**Additional file 2: Table S1.** Z-score ranking comparison between ambulatory and hospitalized patients

## Data Availability

All the data are available form the corresponding author on reasonable request.

## Abbreviations

BMD: Bone mineral density
BMI: Body mass index
DEXA: Dual energy x-ray absorptiometry
FRAX: Fracture risk assessment tool
GC: Glucocorticoid
OR: Odds ratio
PPI: Proton pump inhibitor
SD: Standard deviation
