# Supplementary figures and images for: High detection rate of osteoporosis with screening of a general hospitalized population: a 6-year study in 6406 patients in a university hospital setting
Source: BMC Musculoskelet Disord. 2020 Feb 10;21:90. doi: 10.1186/s12891-020-3116-9 (PMC7011267; doi:10.1186/s12891-020-3116-9)

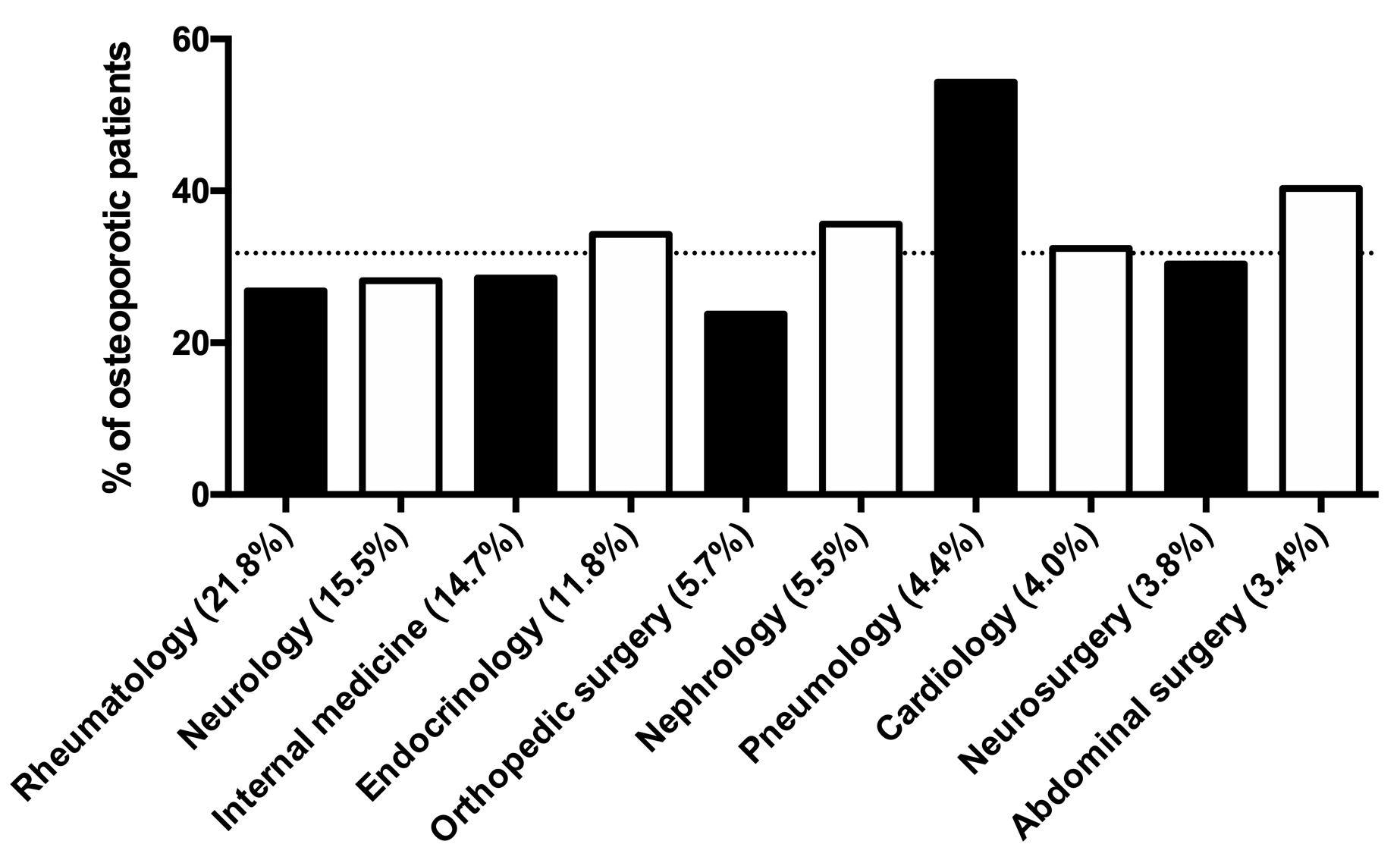

Supplement: Supplementary file 1 — Additional file 1: Figure S1. Percentage of osteoporotic patients by department in the hospitalized sub-population. The horizontal line represented the mean percentage in the hospitalized sub-population. The percentage in the X axe legend after the name of the department represent the % of DEXA performed by the department regards to the total number of DEXA performed in the hospitalized sub-population. [file 12891_2020_3116_MOESM1_ESM.tiff]
