# Supplementary material for: High detection rate of osteoporosis with screening of a general hospitalized population: a 6-year study in 6406 patients in a university hospital setting
Source: BMC Musculoskelet Disord. 2020 Feb 10;21:90. doi: 10.1186/s12891-020-3116-9 (PMC7011267; doi:10.1186/s12891-020-3116-9)
Supplement: Supplementary file 2 — Additional file 2: Table S1. Z-score ranking comparison between ambulatory and hospitalized patients [file 12891_2020_3116_MOESM2_ESM.docx]

**SUPPLEMENTARY DATA**

|  | | Ambulatory patients | | Hospitalized patients | |  |
| --- | --- | --- | --- | --- | --- | --- |
|  | Categories | N | Number (%) | N | Number (%) | p-value |
| Z-score lumbar spine < -1 |  | 3228 |  | 1334 |  | **<0.0001** |
|  | No |  | 2645 (81.9) |  | 1018 (76.3) |  |
|  | Yes |  | 583 (**18.1**) |  | 316 (**23.7**) |  |
| Z-score femoral neck < -1 |  | 3179 |  | 1314 |  | **<0.0001** |
|  | No |  | 2708 (85.2) |  | 1005 (76.5) |  |
|  | Yes |  | 471 (**14.8**) |  | 309 (**23.5**) |  |
| Z-score total hip < -1 |  | 3184 |  | 1311 |  | **<0.0001** |
|  | No |  | 2695 (84.6) |  | 968 (73.8) |  |
|  | Yes |  | 489 (**15.4**) |  | 343 (**26.2**) |  |
| Z-score lumbar spine < -2 |  | 3228 |  | 1334 |  | **<0.0001** |
|  | No |  | 3067 (95.0) |  | 1191 (89.3) |  |
|  | Yes |  | 161 (**5.0**) |  | 143 (**10.7**) |  |
| Z-score femoral neck < -2 |  | 3179 |  | 1314 |  | **<0.0001** |
|  | No |  | 3110 (97.8) |  | 1246 (94.8) |  |
|  | Yes |  | 69 (**2.2**) |  | 68 (**5.2**) |  |
| Z-score total hip < -2 |  | 3184 |  | 1311 |  | **<0.0001** |
|  | No |  | 3094 (97.2) |  | 1217 (92.8) |  |
|  | Yes |  | 90 (**2.8**) |  | 94 (**7.2**) |  |
| Z-score lumbar spine < -2.5 |  | 3228 |  | 1334 |  | **<0.0001** |
|  | Non |  | 3154 (97.7) |  | 1265 (94.8) |  |
|  | Oui |  | 74 (**2.3**) |  | 69 (**5.2**) |  |
| Z-score femoral neck < -2.5 |  | 3179 |  | 1314 |  | **<0.0001** |
|  | Non |  | 3159 (99.4) |  | 1284 (97.7) |  |
|  | Oui |  | 20 (**0.6**) |  | 30 (**2.3**) |  |
| Z-score total hip < -2.5 |  | 3184 |  | 1311 |  | **<0.0001** |
|  | Non |  | 3158 (99.2) |  | 1271 (96.9) |  |
|  | Oui |  | 26 (**0.8**) |  | 40 (**3.1**) |  |

Table S1. Z-score ranking comparison between ambulatory and hospitalized patients.
